# Spinal Hydatidosis Relapse: A Case Report

**DOI:** 10.1155/2014/207643

**Published:** 2014-07-21

**Authors:** Roberto Fiori, Irene Coco, Marco Nezzo, Gisèle Kabunda, Giuseppe Emmanuele Umana, Mario Francesco Fraioli, Giovanni Simonetti

**Affiliations:** ^1^Department of Diagnostic Imaging and Interventional Radiology, Molecular Imaging and Radiotherapy, Fondazione Policlinico “Tor Vergata”, Viale Oxford 81, 00133 Rome, Italy; ^2^Department of Neurosurgery, University of Rome Tor Vergata, Viale Oxford 81, 00133 Rome, Italy

## Abstract

Human cystic echinococcosis (CE) is a zoonosis caused by the larval stage of the *Echinococcus granulosus* and the most common sites affected are the liver and lung in approximately 80–90% of cases. The hydatid bone represents the 0.5–2.5% of all cases and localization cord is present about 50% of the time. This benign and commonly asymptomatic disease may simulate an aggressive malignancy because of osseous destruction and aggressive extension. We report a case of a 42-year-old male patient, presented with an unusual spinal hydatidosis relapse, related to anthelmintic drug therapy withdrawal after 10-year treatment. The man had previous excision of chest and hepatic hydatid cysts (resp., 10 and 3 years ago) and after primary mediastinal and spinal involvement (3 years ago) he was lost to follow-up and discontinued drug therapy. The patient underwent surgery and the postoperative histopathology confirmed the diagnosis. The patient recovered with no complications. Despite significant progress in diagnostic imaging, pharmacological and surgical therapy, spinal CE remains associated with high morbidity.

## 1. Introduction

Hydatid disease is an important infestation caused by the parasite* Echinococcus granulosus *and still common in countries in the temperate zones, including the Mediterranean countries, the Middle East, South America, New Zealand, Australia, and Southeast Asia and China [1, 2].

The cestode's lifecycle involves two hosts. The definitive host is usually the dog while the humans are the incidental intermediate hosts and can become infected by the ingestion of the eggs of the parasite that commonly shed in the feces of canids.

Larvae emerge from the eggs in the intestine; and after invasion to the blood vessels, they migrate into almost every part of the body [3].

In the accidental human intermediate host, the characteristic cystic lesions are mainly found in the liver (~70%) and the lungs (~20%), but virtually any part of the body may be affected, including the bone (~0.5–4%). The central nervous system (which is involved in ~3% of all cases) and the vertebral column (which is involved in ≥50% of the ~0.5–4% of cases affecting the bone) [4, 5] are particularly vulnerable given the sequelae that result from their involvement.

This disease has no specific characteristics as cord compression and existence of multiple organ hydatidosis. Positive hydatid immunology test may support the diagnosis, but negative result may not exclude the disease [4].

The spinal hydatid disease is a rare cause of neurological signs and symptoms and should be considered in the differential diagnosis of spinal cord compression syndrome in endemic areas. There are no characteristic signs or symptoms and misdiagnosis is easily to be made preoperatively. Most diagnoses are made intraoperatively, which increases the risk of future recurrences [6]. It has recurrence rates ranging from 30% to 100% despite anthelmintic therapy and aggressive surgical treatment.

Spinal hydatidosis (involvement of the spinal cord, the spine, or both structures) occurs in 1% of all cases of CE and is most commonly located in the dorsal spine [7] and cord involvement is present about 50% of the time. The treatment of choice is surgical, with removal of the intact cysts being of vital importance. Perforation of the cysts during an operation may lead to systemic dissemination and more critically to local seeding which results in chronic recurrence. Curative surgery remains difficult with bone involvement as infiltration of the bone hampers unruptured and complete resection of the cysts and high recurrence rates plague the long-term outcome [8].

Besides surgery, the only other treatment option for spinal CE is antiparasitic therapy with benzimidazole compounds. Since the introduction of mebendazole (in the 1970s) and albendazole (in the early 1980s), surgery with concomitant and subsequent benzimidazole administration became the widely accepted treatment standard and in cases where surgery is not possible, these drugs remain the only treatment option [9].

We discuss a case of a 42-year-old patient with cervicodorsal spinal CE relapse, after primary spine surgical intervention; his symptoms remained under control over the following 3 years, whereupon he refused taking anthelmintic drug therapy with a recurrence and rapid progression of neurological deficits.

## 2. Case Report

We present a case of a 42-year-old man already operated 10 years ago for CE of the liver and 3 years ago for mediastinal and primary spinal disease involvement.

He comes to our attention in 2010 after access to our emergency department for progressive paraparesis since 1 week with inability to walk, level of anesthesia below D2 associated with worsening and marked sphincter disorders, and pain associated with cervical-dorsal refractory to analgesic therapy.

**Figure 1:**
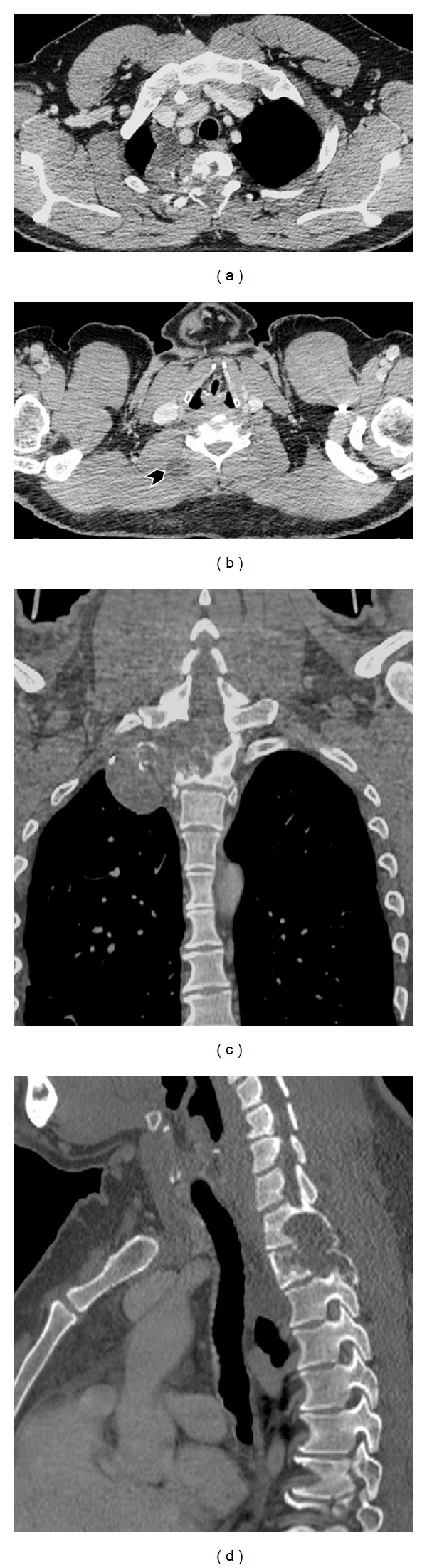
CT exam after intravenous contrast injection: axial (a) and (b), coronal (c), and sagittal (d) planes demonstrate cystic tissue localized in the chest and in the paravertebral muscles on the right (arrow head) and bone erosion of second rib and D1 and D2 vertebrae.

Computed tomography (CT) (Figure 1) demonstrated cystic tissue localized to the mediastinum and the paravertebral muscles with the erosion of the second rib and the vertebral bodies of D1 and D2.

**Figure 2:**
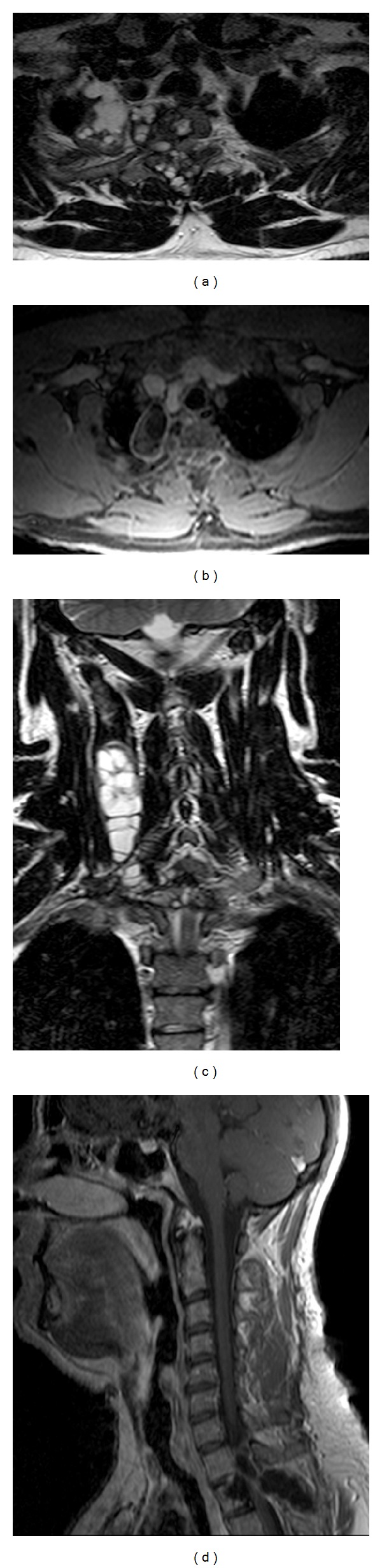
T2 images (a) and (b) show a cystic inhomogeneous tissue localized in the chest and the paravertebral muscles on the right that erodes second rib and D1 and D2 vertebrae compressing the spinal cord at that level. T1 after gadolinium intravenous injection images (c) and (d) demonstrates a predominantly cystic component that presents peripheral enhancement after contrast medium.

**Figure 3:**
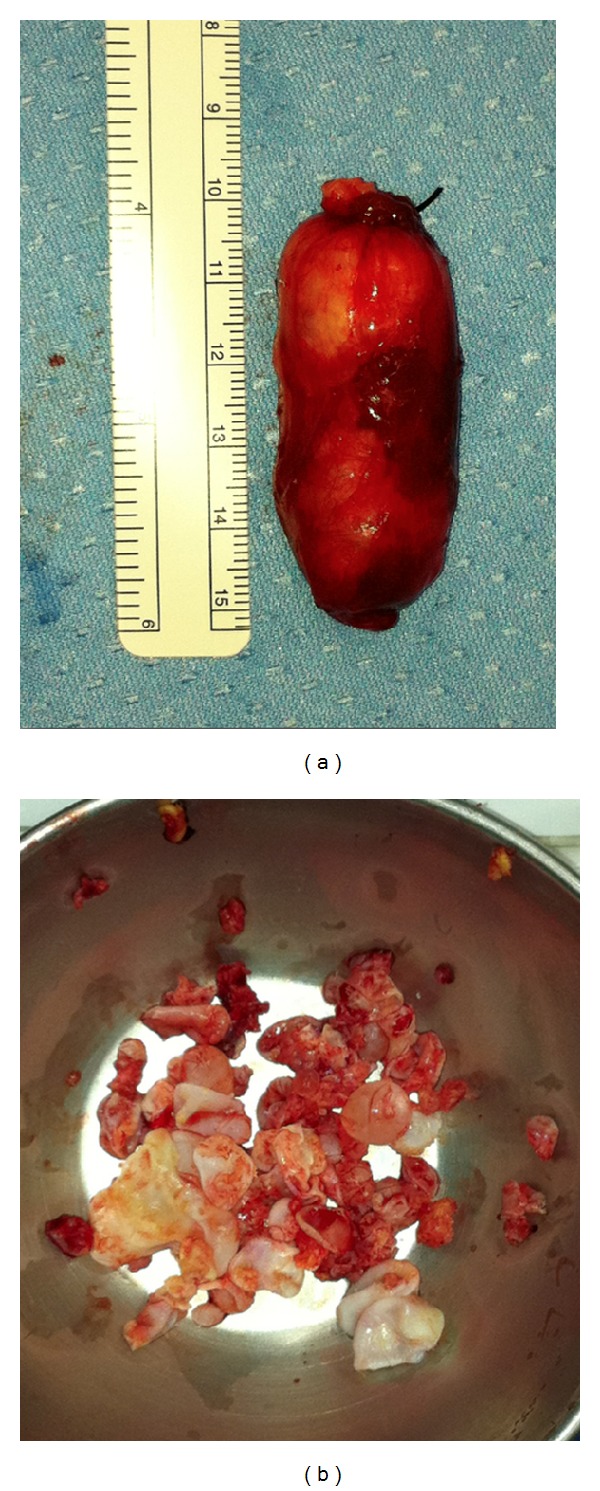
(a) Single hydatid training (2 × 5 cm) and (b) multiple hydatid cysts removed.

**Figure 4:**
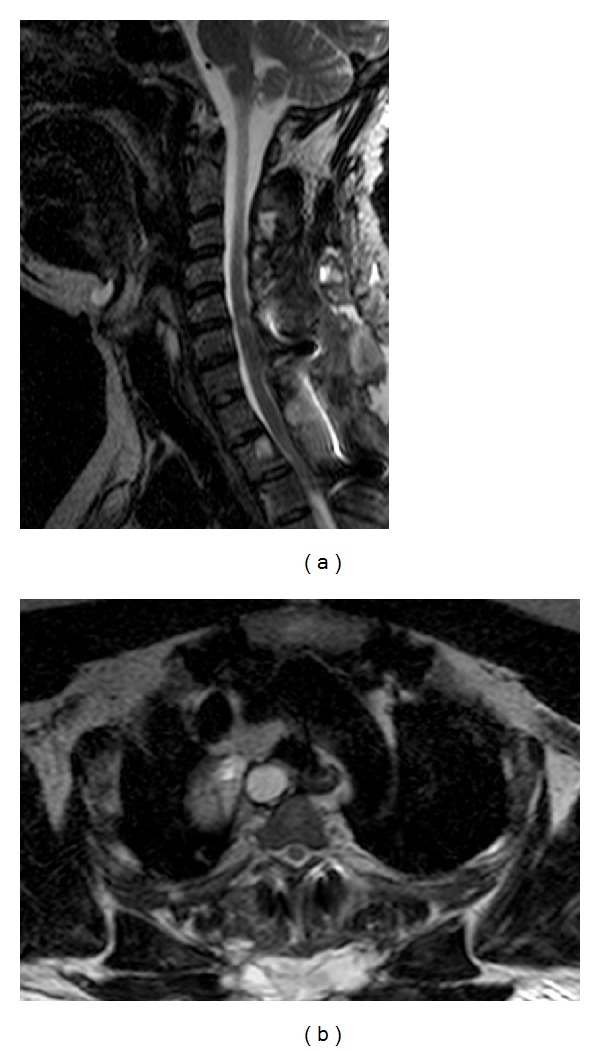
T2 images sagittal and axial planes (a) and (b) show the placement of a vertebral spacer, the successful decompression of the spinal cord, and a residual tissue in the chest on the right.

Cervicodorsal spine MRI with intravenous injection of gadolinium (Figure 2) showed multiple and heterogeneous lesions located in the intraspinal extradural space at D1-D2 level, with severe spinal cord compression and involvement of the transverse processes, spinous processes, the left laminae, the second rib, the mediastinum on the right side, and cervical paraspinal muscles from C4 to D2. The patient was subsequently treated with an anthelmintic drug (Albendazole) and steroid therapy for the neurologic deficit. The patient underwent a first surgical approach by right sided paramedian longitudinal incision from C4 to D2 with the removal of the hydatid cyst (2 × 5 cm) located within the paraspinal muscle (Figure 3(a)); the second surgical approach was performed in the same surgical procedure to remove numerous smaller hydatid cysts (Figure 3(b)) with a median posterior approach, performing decompressive laminectomy and spinal stabilization from C6 to D4 with Universal Clamp Spinal Fixation System-Zimmer (Figure 4).

MRI performed after surgery showed the persistence of a small cyst located in the body of D2. It was decided with the thoracic surgeon to remove the small residual cyst during surgery for the removal of mediastinal cysts, with an anterior approach; however, the patient refused the thoracic surgery.

Three years later (in 2013) after he voluntary stopped medical treatment, the patient develops a rapid worsening of spinal deficits with motor deficit predominantly on the right side with progressive paraparesis, neuralgiform distal pain, marked sphincter disorders, and lack of peripherical sensitivity.

**Figure 5:**
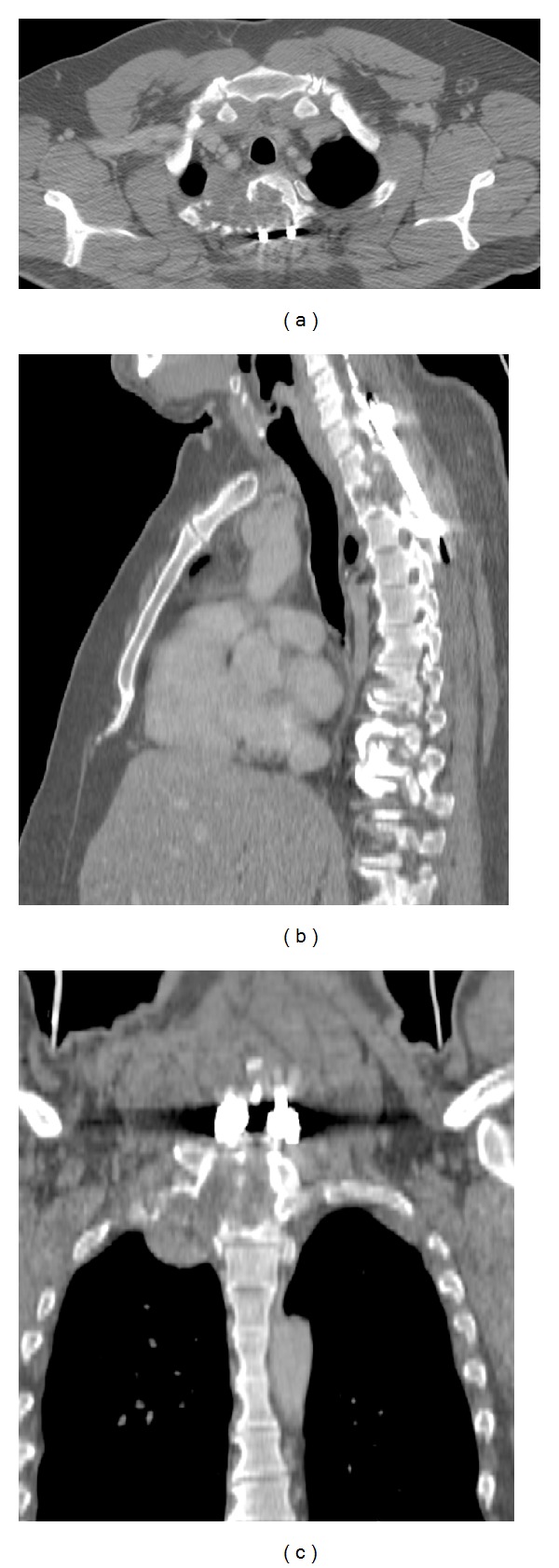
CT exam axial, sagittal, and coronal planes (a), (b), and (c) reveal a recurrence at the level of D1 and D2 vertebrae and the presence of cystic tissue in the chest on the right.

**Figure 6:**
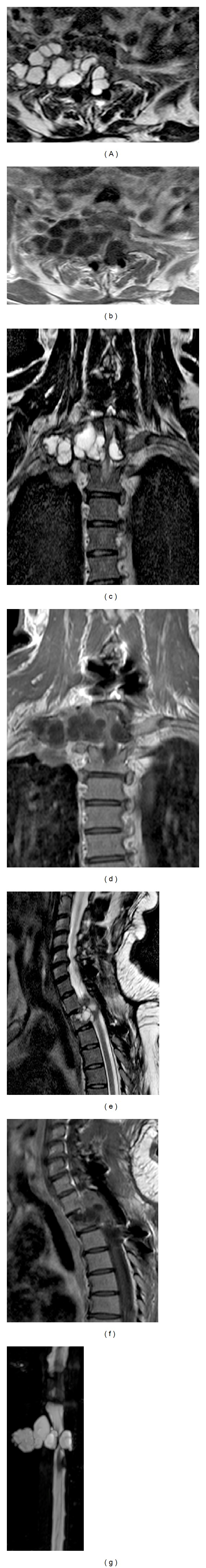
T2 images (a), (c), and (e), T1 images after intravenous gadolinium injection (b), (d), and (f), and MR myelography (g) reveal the presence of a cystic tissue in the chest on the right and severe spinal cord compression due to the presence of multiple hydatid cysts involving the body of D2 and minimally the body of D1.

CT (Figure 5) and MRI with intravenous injection of gadolinium (Figure 6) showed a recurrence of multiple intraspinal extradural cystic lesions, with the spinal cord compression involving the body of D2 and minimally the body of D1. The patient will undergo a second surgical procedure of decompression of the spinal cord, removing the recurred cysts and maintaining the spinal stabilization system. The patient recovered successfully and was able to walk autonomously on the fifth day after surgery. Later the patient began treatment with anthelmintic drug (Albendazole), with stability of the pathology until today.

## 3. Conclusions

In postoperative time, the neurological symptoms progressively and markedly regressed and the patient recovered strength in the lower limbs to ambulate independently recovering after 10 days. They, too, improved the sensory deficit that the sphincter disorders, being able to remove the catheter.

Based on the experience presented, drug anthelmintic therapy is safe and must be considered as a lifelong treatment for severe case of spinal hydatidosis [10] even because the possibilities for a radical treatment are poor [11], and the important findings are the following criteria: diagnosis and appropriate preoperative medical treatment, intraoperative surgical devices, and the knowledge that particular case; however, in the occurrence of bone hydatidosis recurrence is frequent, so a single surgery may not be sufficient to control the disease.

“*Prognosis in vertebral hydatid disease is almost hopeless as regards complete cure. This is due to the impossibility of removing by surgical means all diseased bone, especially if the vertebral bodies are affected, to the multiplicity of extra-osseous cysts, to the certainty of recurrent pressure on the cord*…” (Dew, 1928) [12].
